# Maternal Health: Time to Deliver

**DOI:** 10.1371/journal.pmed.1000300

**Published:** 2010-06-21

**Authors:** The PLoS Medicine Editors

## Abstract

The *PLoS Medicine* editors argue that maternal health policies must recognize the full spectrum of women's sexual and reproductive health.

A shocking number of women die or are disabled each year trying to give birth—more than 10 million women, according to the WHO, 99% of whom are in developing countries [1]. The maternal health Millennium Development Goals (MDGs)—to reduce maternal mortality by three-quarters and to provide universal access to reproductive health by 2015—are long considered the most underachieving of the MDGs and have been chronically low on political priority lists. Recent international attention to maternal health, including its prominence at upcoming G8, Pacific Health, African Union, and UN General Assembly Summits, is therefore a welcome development and comes at a time when critical new data are available to guide action.

This month *PLoS Medicine* releases new data on priority interventions for maternal health, part of a series on maternal, neonatal, and child health (MNCH) in Africa. The goal of the series, authored by a group of international collaborators, is to identify scientific interventions and accelerate actions that are tailored and specific to sub-Saharan Africa. Based upon consultations with 60 scientists and policy makers from nine countries and using local and national data, the series updates the current status of MNCH in the region, reviews evidence based solutions, and identifies high-impact opportunities for reducing maternal and child mortality. Their data on maternal health are illuminating, particularly for maternal mortality (defined as any death of a woman during pregnancy or in the 42 days after). But the series also highlights the fact that, for genuine progress on maternal health to be realized, policy agendas must include recognition of the full spectrum of women's sexual and reproductive health.

In the first paper in the series [2], Joy Lawn and colleagues report that the main sources of maternal death in sub-Saharan Africa are related to direct obstetric complications including infection, hemorrhage, eclampsia, and obstructed labor; these amount to 64% of the burden. HIV/AIDS, pneumonia, and malnutrition also contribute. Maternal mortality in sub-Saharan Africa is twice as high among poor women as among less-poor women, and rates are consistently higher among uneducated and rural women, and in areas of conflict. As others have stated, the most important hazards to maternal health, including unsafe abortion, are preventable with access to proven health care [1].

The analysis by Lawn and colleagues of access to interventions that are known to improve maternal health reveals good and bad news. Across the region 71% of pregnant women receive at least one antenatal care visit with a skilled attendant such as a doctor, nurse, or midwife. But less than half of all births are attended by skilled personnel, although some countries like Benin, Burkina Faso, and Ghana have made important progress that others can model. Inequity is stark: there is a 5-fold higher rate of skilled attendance at birth among the least poor versus the most poor in most countries. Contraception prevalence rates are only about 23% in the region; but worse, data are not consistently collected nor are these interventions given policy priority, say the authors. Despite reproductive health care being a vital part of the recommended packages of care for mothers and children, the authors report that there are currently no routine indicator data across sub-Saharan Africa for reproductive health services such as post-abortion care and family planning, or for family and community care such as adolescent and pre-pregnancy nutrition and prevention of sexually transmitted infections including HIV in pregnancy.

The second paper in the series [3], by Robert Black and colleagues, reports the results of a priority setting analysis using the Lives Saved Tool (LiST). Estimates of mortality reduction for 42 sub-Saharan African countries shows that nearly 4 million deaths among women, newborns, and children would be averted if already well known interventions such as emergency obstetric care, breastfeeding counseling, and treatment for diarrhea and pneumonia were to reach 90% of families. A detailed analysis of nine diverse countries estimated mortality reductions and additional cost for feasible increases in coverage (to 20%) of high-impact interventions. Among those findings directly related to maternal health, Black and colleagues report that even in areas with the weakest health systems, such as Ethiopia and northern Nigeria, the increased use of contraceptives could avert a quarter of maternal deaths each year and would cost only an additional 17 cents (US) per capita.

The *PLoS Medicine* series relies upon new statistics on maternal mortality that demonstrate the ongoing scale of the problem and the particular urgency for action within sub-Saharan Africa. Using updated mortality data for 181 countries and sophisticated statistical techniques, Christopher Murray and colleagues reported an overall reduction in the number of women dying from pregnancy and childbirth each year from 526,300 in 1980 to about 342,900 in 2008 [4]. Lowered pregnancy rates, higher incomes (which improve nutrition and access to health care), more education for women, and better availability of skilled attendants at birth are said to be responsible. Important variations across and within regions are masked by the aggregate gains: while large declines in maternal mortality occurred in central Europe and south Asia (by as much as two-thirds since 1980), in all regions of sub-Saharan Africa rates have increased in the 1990s and progress since has been negligible [2]. Moreover, the proportion of global maternal deaths that occurred in sub-Saharan Africa nearly doubled: from 23% in 1980 to 52% in 2008. The contribution of HIV, a particular concern in Africa, demands specific attention: without the HIV epidemic there would have been an estimated 61,400 fewer maternal deaths worldwide in 2008.

Sadly, these new analyses confirm the vulnerability and neglect of African women. The *PLoS Medicine* series also affirms the frustrating fact that we already know what works for reducing maternal deaths. Much of the burden of maternal mortality is routinely preventable with known, cost-effective interventions that those of us in the developed world take for granted: good nutrition, antenatal care, skilled attendance at delivery, emergency obstetric care, and family planning. It is unconscionable that poor coverage, poor quality, and inequities in the provision of these essential MNCH interventions persist in sub-Saharan Africa. With five years left to achieve the maternal health MDGs, evidence-based action in Africa must be built upon the identification and implementation of these priority interventions.

But while these new data are valuable for advancing the selected interventions, they also reveal the importance of considering the whole of women's reproductive and sexual health in maternal health policy. Contraception access rates continue to be unacceptably low, consistent with previous estimates of 215 million women worldwide having unmet needs for contraception [4]. That increased use of contraceptives—an exceptionally low cost intervention—could avert a quarter of maternal deaths each year in even in the most under-resourced health systems demands action on this front.

Indeed, action on maternal health will do well to focus on increasing access to family planning resources together with basic medical care. Family planning—the ability to choose the number, spacing, and timing of children—is vital to educational attainment and economic productivity, and reducing unwanted pregnancies and family size is associated with lower public spending on the health care system and social services [5]. Further, access to family planning services, including contraception and abortion, forms the basis of women's empowerment and control over their sexual and reproductive lives and is vital to women's health and dignity [5],[6]. Recognition of women's sexual and reproductive rights is particularly essential to maternal health progress in regions where women's status is diminished and their inability to negotiate safe sex increases the risk of leading causes of death and disability such as HIV, other sexually transmitted infections, unplanned pregnancies, and sexual violence.

As global leaders meet this spring and build their 2010 maternal health agendas, these new data can inform an evidence-based approach to policy that can reduce maternal deaths, support women's ability to determine their reproductive and sexual lives, increase access to family planning resources, and ensure that women are no longer denied the benefits of even the most basic medical care during pregnancy and childbirth. It's time to deliver on promises to improve maternal health.

## References

[1] WHO (2005). The world health report 2005 – Make every mother and child count.. http://www.who.int/whr/2005/en.

[2] Kinney MV, Kerber KJ, Black RE, Cohen B, Nkrumah F (2010). Sub-Saharan Africa's Mothers, Newborns, and Children: Where and Why Do They Die?. PLoS Med.

[3] Friberg IK, Kinney MV, Lawn JE, Kerber KJ, Odubanjo MO (2010). Sub-Saharan Africa's Mothers, Newborns, and Children: How Many Lives Could Be Saved with Targeted Health Interventions?. PLoS Med.

[4] Hogan MC, Foreman KJ, Naghavi M, Ahn SY, Wang M (2010). Maternal mortality for 181 countries, 1980—2008: a systematic analysis of progress towards Millennium Development Goal 5.. Lancet.

[5] Singh S, Jarroch JE, Vlassof M, Nadeau J (2003). Adding It Up: The Costs and Benefits of Investing in Family Planning and Maternal and Newborn Health, New York: Guttmacher Institute and United Nations Population Fund, 2009.. http://www.guttmacher.org/pubs/addingitup.pdf.

[6] Amnesty International (2009). Demand Dignity: Dying too young: Maternal mortality claims the life of one woman every minute.. http://www.amnesty.org/en/library/info/ACT35/005/2009/en.

